# Intrahepatic biliary cystadenoma mimicking hydatid cyst of liver: a clinicopathologic study of six cases

**DOI:** 10.1186/s13256-017-1481-2

**Published:** 2017-11-10

**Authors:** Zubair Ahmad, Nasir Uddin, Wasim Memon, Jamshid Abdul-Ghafar, Arsalan Ahmed

**Affiliations:** 1 Department of Pathology and Laboratory Medicine, Aga Khan University Hospital, Karachi, Pakistan; 2 Department of Radiology, Aga Khan University Hospital, Karachi, Pakistan; 3 Department of Pathology and Laboratory Medicine, French Medical Institute for Mothers and Children (FMIC), Behind Kabul Medical University Aliabad, P.O. Box: 472, Kabul, Afghanistan

**Keywords:** Biliary cystadenoma, Liver, Cyst, Hydatid cyst, Internal septations

## Abstract

**Background:**

Intrahepatic biliary cystadenomas are rare hepatic neoplasms, which are usually cystic. These tumors are often misdiagnosed as simple liver cysts and hydatid cysts clinically and radiologically owing to nonspecific clinical and radiologic features. These tumors require complete resection, as recurrence and malignant transformation can occur following incomplete excision. It is essential that these tumors be diagnosed accurately so that they can be adequately excised.

**Methods:**

Clinical and radiological features of six cases of biliary cystadenoma are described.

**Results:**

All of these cases were resected with the clinical and/or radiological impression of simple liver cysts and/or hydatid cysts. Out of the six patients, five were female and one was male. Ages of the patients ranged from 28 to 60 years (mean 45 years). The patients presented with nonspecific symptoms. Internal septations were seen on preoperative imaging (when available). On gross examination, all tumors were cystic; their sizes varied from 5.5 to 14 cm, mean size was 9.0 cm. On histopathologic examination, cystic spaces were lined by cuboidal to columnar mucin-secreting epithelium with underlying ovarian-type stroma. In one case, ovarian-type stroma was not seen. Recurrence was seen in three cases at 1 to 5 years of follow up.

**Conclusions:**

Owing to their malignant potential and high recurrence rate following incomplete resection, an aggressive surgical approach is recommended. Prognosis is excellent after complete resection.

## Background

Intrahepatic biliary cystadenomas are rare cysts forming hepatic neoplasms, which are usually multilocular. Clinical, radiological, and histological features are nonspecific and often lead to misdiagnosis. Some tumors may be unilocular. Cysts are usually thin walled and filled with thin watery fluid. In some cases, the cyst wall may be thickened, filled with thicker mucinous fluid and may show papillary projections. They range in size from 2.5 to 28.0 cm. On histopathologic examination, the cystic spaces are lined by cuboidal to columnar mucin-secreting epithelium and ovarian-type stroma is seen subepithelially [1, 2]. However, ovarian-type stroma may not be seen in some cases in male patients [3]. The typical densely cellular ovarian-type stroma is a feature that hepatobiliary cystadenomas share with their counterparts in the pancreas. This distinctive stroma is exclusively present in women and is immunoreactive for estrogen and progesterone receptors as well as for alpha-inhibin [4]. These tumors are much more common in females and most patients are young to middle aged [5–8]. Patients often present with nonspecific abdominal discomfort, although some may present with more specific symptoms, such as jaundice [9, 10]. These tumors are often misdiagnosed as simple liver cysts clinically and even after exhaustive imaging studies such as ultrasound, computed tomography (CT) scan, and endoscopic retrograde cholangiopancreatography (ERCP) [5, 8, 9, 11]. In countries where hydatid cysts are endemic, they are often clinically and radiologically misdiagnosed as hydatid cysts [11–14]. Tuberculosis of the liver, a rare entity, can also mimic biliary cystadenomas on radiological examination [15]. Due to their nonspecific signs and symptoms and their ability to mimic infectious diseases such as hydatid disease, and the resultant misdiagnosis, treatment is often inadequate. These tumors require complete resection because recurrence and even malignant transformation can occur following incomplete excision. Malignant cases often show large papillary masses. It is essential to diagnose these tumors accurately so that they can be adequately excised. Preoperative imaging that demonstrates the presence of internal septations is highly suggestive of biliary cystadenomas [15]. Owing to their malignant potential and high recurrence rate following incomplete resection, an aggressive approach is recommended [5, 6, 9]. Prognosis is excellent after complete surgical resection [7, 16].

Here we present six cases of biliary cystadenoma diagnosed in the Section of Histopathology, Aga Khan University Hospital (AKUH), Karachi, over a 7-year period from 2008 to 2015. All of these cases were resected with the clinical and radiological impression of simple liver cysts or hydatid cysts.

## Methods

We searched the Surgical Pathology files of the Department of Pathology and Laboratory Medicine, AKUH, for cases reported as intrahepatic biliary cystadenomas. The principal authors (NU and ZA) reviewed the slides of all six cases reported as intrahepatic biliary cystadenomas. The clinical and follow-up data were obtained from medical records, hospital discharge summaries, and by telephone inquiry. All available hematoxylin and eosin (H&E), histochemical stains, and immunohistochemical (IHC) stained slides were reviewed and reassessed.

## Results

A total of six cases of intrahepatic biliary cystadenomas were reported between 2008 and 2015. Five patients were from Pakistan (2 Sindhis, 2 Punjabis and 1 Balochi) and one patient was Afghan from Afghanistan. Out of the six cases, five were diagnosed in females and one was diagnosed in a male. Ages of the patients ranged from 28 to 60 years. Mean and median ages were 45 and 43 years respectively. All patients presented with abdominal pain and swelling. In five out of six cases, clinical suspicion was that of a hydatid cyst. In all cases, the lesions had been excised and sent for histopathologic examination.

On gross examination, all tumors were cystic, sizes varied from 5.5 to 14.0 cm with a mean size of 9.0 cm. In two cases, the specimens were received in pieces. The cysts were multiloculated in four and uniloculated in two cases. Cyst wall thickness ranged from 0.3 to 0.7 cm. In all six cases, outer surfaces were smooth, gray white to tan brown in color, shiny and glistening to dull, and firm in consistency. No solid or nodular areas were seen in the wall in any of the cases. Cysts were already opened when they were received in the histopathology laboratory.

### Case 1

A 45-year-old Sindhi woman from Pakistan presented with epigastric and right hypochondrial pain of 2 months’ duration. The clinical impression was that of a hydatid cyst. Radiological films were not available for correlation. She underwent excision of liver cyst in September 2008. The specimen was received as multiple, irregular dull, gray brown pieces measuring 11.0 × 8.0 cm in aggregate. On sectioning, the wall was 0.7 cm in thickness; the cut surface was firm in consistency and gray with patchy areas of hemorrhage. Solid or nodular areas were not seen. Histopathologic examination revealed a cyst wall lined by a single layer of tall columnar epithelial cells with basally placed uniform nuclei and apical mucin, highlighted on periodic acid–Schiff +/− Alcian blue (PAS+/−AB). Dysplasia was not seen. Stroma was composed of plump spindle cells resembling ovarian stroma. The epithelial cells showed positivity for IHC stain cytokeratin (CK) AE1/AE3. A diagnosis of biliary cystadenoma was made. As the specimen was received in pieces, comments on excision margins could not be made. The patient was lost to follow up.

### Case 2

A 43-year-old Punjabi woman from Pakistan presented with abdominal pain and fullness of several months’ duration. Clinical diagnosis was hydatid cyst of the liver. Radiological films were not available for correlation. The cyst was excised in December 2010. The specimen was received as multiple flattened pieces, tan in color, and firm in consistency, measuring 5.5 × 2.0 cm in aggregate. The cut surface was firm, tan brown in color, and 0.5 cm in thickness. Solid or nodular areas were not seen. The microscopic sections showed a cyst wall lined by a single layer of tall columnar epithelium with basally placed nuclei and apical mucin highlighted by special stain (PAS+/−AB). Dysplasia was not seen. Epithelial cells were positive for CK AE1/AE3 and CK 7. Stroma was compact and spindly resembling ovarian stroma and showed positivity for the stain anti-smooth muscle actin (ASMA). A diagnosis of intrahepatic biliary cystadenoma was made. As the specimen was received in pieces, comments on excision margins could not be made. The patient developed recurrence in late 2015, almost 5 years after the initial surgery. She underwent repeat resection in December 2015.

### Case 3

**Fig. 1:**
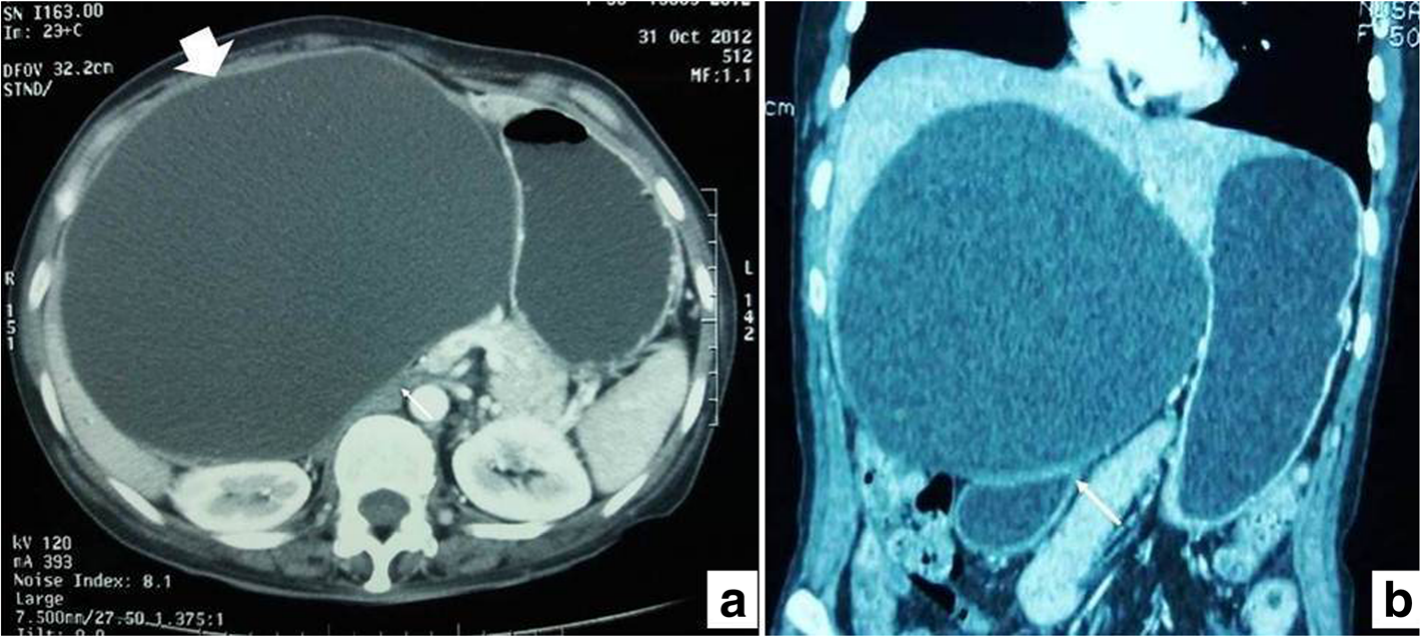
Axial and coronal sections from contrast-enhanced computed tomography of the abdomen. **a** There is a well-circumscribed, intrahepatic cystic lesion having thin walls (*bold arrow*). There are no internal septations, mural nodules, or abnormal wall enhancement, favoring diagnosis of cystadenoma. **b** There is significant associated mass effect resulting in compression over duodenum (*thin arrow*)

A 50-year-old Afghan woman from Afghanistan presented with abdominal pain and swelling in her right hypochondrium. The clinical differential diagnosis included simple liver cyst and hydatid cyst. A CT scan of her abdomen and pelvis showed a large cystic lesion in her right upper abdomen measuring 17.0 × 17.0 × 15.0 cm in dimensions. It was pushing the hepatic flexure inferiorly and right kidney posteriorly and abutting the head of her pancreas and the anterior abdominal wall anteriorly. It was abutting the right lateral abdominal wall on the right side and lesser curvature of her stomach on the left side. It demonstrated few enhancing septa. No solid areas or calcification were seen. It was difficult to determine the exact site of origin. However, it seemed to be arising from her liver with exophytic growth in a downward direction. Her intrahepatic ducts were not dilated. The cystic mass was pushing her portal vein upward. Her portal vein was however normal in diameter. The mass was pushing her gallbladder and her right kidney downward (Fig. 1). Differential diagnoses of hydatid cyst of the liver and large necrotic abscess were given. She underwent excision of “liver cyst” in November 2012. The specimen was received as a single, flattened, irregular, firm tissue piece, which appeared to be part of a cyst wall. It measured 4.5 × 3.5 cm in diameter and was 0.4 cm in thickness. The outer surface was gray white and shiny. The inner surface was tan brown with hemorrhagic areas. Solid or nodular areas were not seen. The microscopic sections showed a cyst wall lined by a single layer of mucinous columnar epithelium with subepithelial spindly ovarian-type stroma. There was no dysplasia. The acid mucin in the epithelial cells was highlighted on mucin stain (PAS+/−AB); epithelial cells and stroma were positive for IHC stains CK AE1/AE3 and ASMA respectively. A diagnosis of intrahepatic biliary cystadenoma was made. She remains free of recurrent disease.

### Case 4

A 38-year-old Sindhi woman from Pakistan presented with abdominal pain and fever. The clinical differential diagnosis included pancreatic pseudocyst, liver abscess, and hydatid cyst of the liver. A radiological examination showed a hypodense, well-defined, fluid attenuation lesion in the region of the porta hepatis, involving segments V and VI of her liver, with thin internal septations and no post contrast enhancement. The cyst was exophytic, measured 6.0 × 6.0 cm, abutted the head of her pancreas, and was displacing her gallbladder. Her portal vein and hepatic veins were normally enhancing. Features of liver abscess or pancreatic pseudocyst were not seen. Her liver was normal in size with normal attenuation and enhancement. A radiological diagnosis of “hydatid cyst” was made. She underwent excision of the cyst in March 2013. The specimen was received for histopathologic examination and it comprised a single, irregular, flattened, firm piece of tissue measuring 2.5 × 2.5 cm. The thickness of the wall was 0.3 cm. The inner surface was smooth. Solid or nodular areas were not seen. Histopathologic examination showed a cyst lined by cuboidal to low columnar epithelium (positive for IHC stains CK 7 and CK AE1/AE3), and subepithelial ovarian-type spindly stroma (positive for ASMA and inhibin). Dysplasia was not seen. A diagnosis of intrahepatic biliary cystadenoma was made. She developed abdominal pain and swelling a few months after the initial excision. Repeat radiology re-demonstrated an exophytic, hypodense, lobulated fluid-attenuated lesion with thin internal septation in the region of the porta hepatis involving segment VI of the liver and measuring 11.0 × 8.0 × 7.0 cm. It was displacing her gallbladder anteriorly. In view of her history, a radiological diagnosis of recurrent intrahepatic biliary cystadenoma was made. She underwent a second excision in December 2013 and we received a cystic tissue piece, flattened and already opened, measuring 11.5 × 6.5 cm. On sectioning, it was multiloculated and the inner surface was coated with dark brown, hemorrhagic material. Representative sections showed cystic spaces lined by cuboidal to columnar epithelium with underlying compact, spindly stroma resembling ovarian stroma. There was extensive hemorrhage, acute and chronic nonspecific inflammation, and fibrosis. Liver tissue was identified at the periphery. A diagnosis of recurrent intrahepatic biliary cystadenoma was made.

### Case 5

**Fig. 2:**
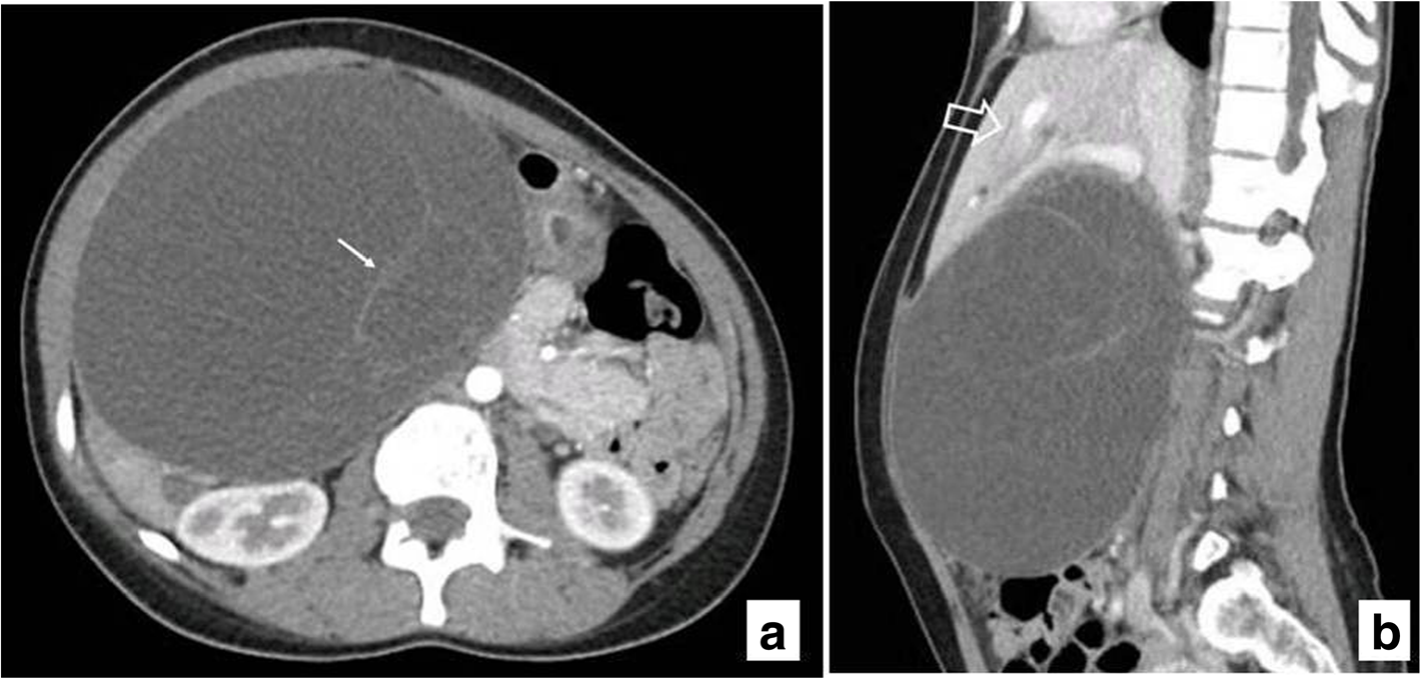
Axial and sagittal sections from computed tomography of the abdomen. **a** There is a large, well-circumscribed, multiloculated, intrahepatic cystic lesion. Fine internal septations without enhancing component are appreciated (*thin arrow*). **b** There is associated mass effect resulting in splaying of branches of portal vein and intrahepatic biliary dilatation (*thick arrow*)

**Fig. 3:**
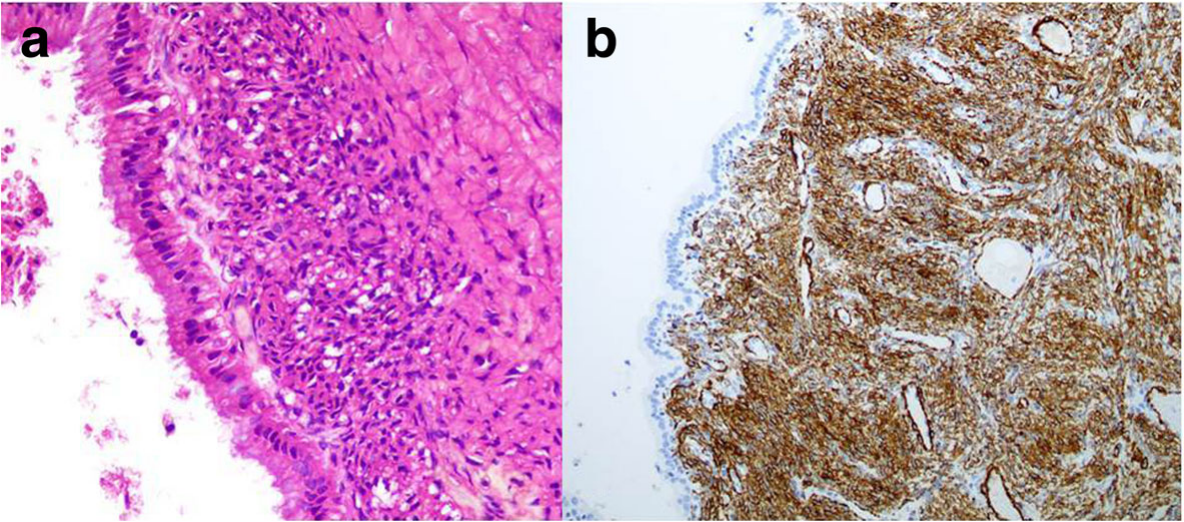
**a** The cyst wall is lined by columnar epithelium with cytoplasmic mucin and subepithelial ovarian-type stroma (hematoxylin and eosin, 400 × magnification). **b** Strong immunoreactivity of anti-smooth muscle actin in ovarian-type stroma

A 28-year-old Balochi woman from Pakistan presented with abdominal pain and swelling. The clinical diagnosis was hydatid cyst of the liver. A radiological examination demonstrated a large, multiseptate, cystic lesion with thin internal septa measuring 14.0 × 12.5 cm arising from the right lobe of her liver. It was splaying the right and left branches of her portal vein. It was causing significant mass effect resulting in compression of her inferior vena cava (IVC), displacement of her aorta as well as stomach and small bowel loops to the left side, and displacement of transverse colon inferiorly and gallbladder posteriorly. Mild intrahepatic biliary dilatation was noted in both lobes of her liver, most likely due to compression effect of the cyst over her common bile duct (CBD; Fig. 2). Radiological diagnosis was hydatid cyst. She underwent surgical resection in February 2015. The specimen was received in the form of a previously opened cyst measuring 14.0 × 13.0 cm. Both outer and inner surfaces were gray brown, roughened, dull, and hemorrhagic. The wall did not show any solid or nodular areas. The cyst was multilocular and average wall thickness was 0.4 cm. Histopathologic examination showed a cyst wall lined by columnar epithelium (highlighted on PAS+/−AB, and positive for CK AE1/AE3) and underlying stroma resembling ovarian stroma (positive for ASMA). The epithelium did not show dysplasia (Fig. 3). A diagnosis of intrahepatic biliary cystadenoma was made. Eight months after initial resection, a repeat CT scan of her abdomen in November 2015 demonstrated a significantly smaller cystic lesion in her liver with thin internal septations measuring 5.0 × 4.0 cm. Radiological diagnosis of recurrent/residual intrahepatic biliary cystadenoma was made. She was scheduled to undergo repeat excision in March 2016 but was then lost to follow up.

### Case 6

**Fig. 4:**
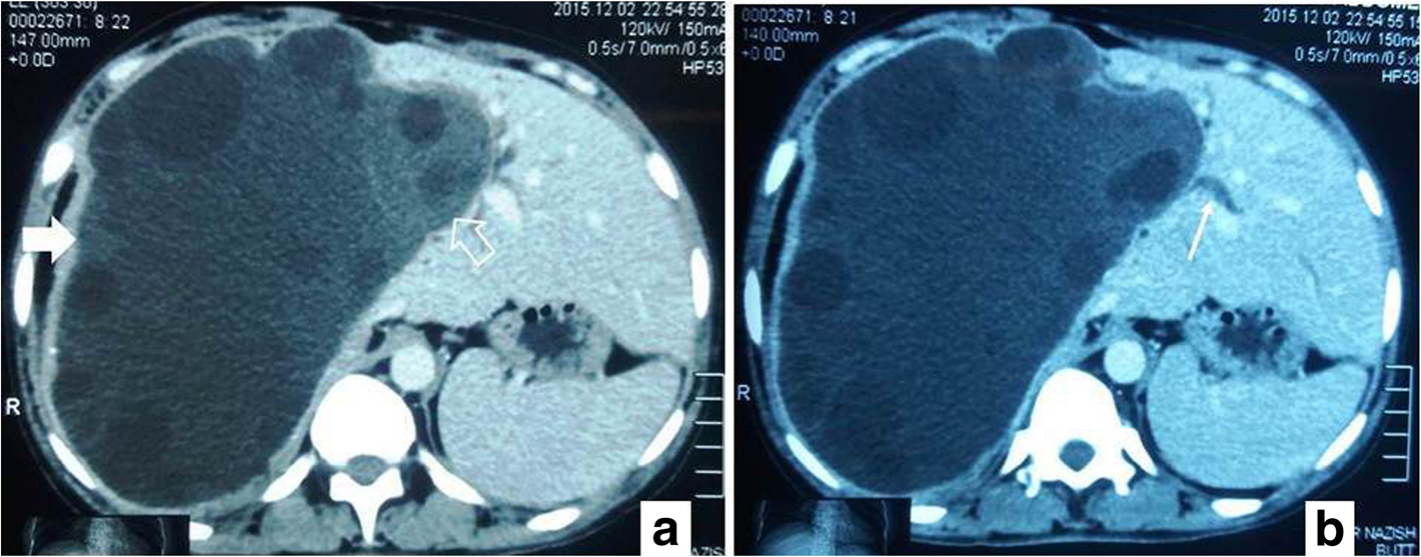
Axial and coronal sections from contrast-enhanced computed tomography of the abdomen. **a** There is a well-circumscribed, multiloculated, intrahepatic cystic lesion (*bold arrow*). The few areas of high attenuation probably represent hemorrhage within the lesion (*thick arrow*). **b** There is significant mass effect with moderate intrahepatic biliary dilatation (*thin arrow*)

**Fig. 5:**
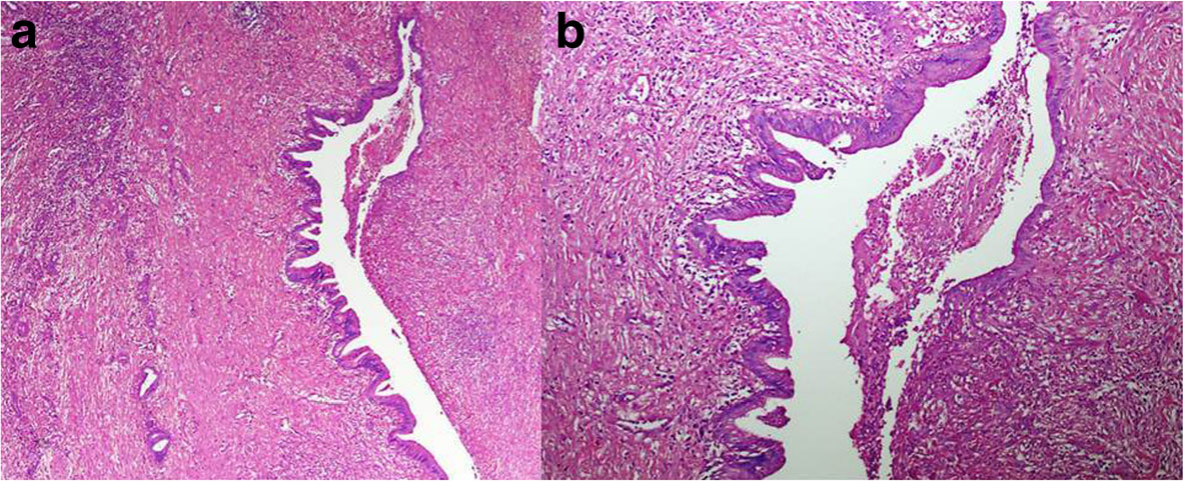
Histology of biliary cystadenoma of the single male patient of the series. **a** The cyst lining is columnar with cytoplasmic mucin. **b** Ulceration of the lining with fibrinopurulent exudate covering. No ovarian-type stroma is seen

A 60-year-old Punjabi man from Pakistan presented with abdominal pain and obstructive jaundice. The clinical impression was of a hydatid cyst. A CT scan of his abdomen revealed a hypodense, well-defined, multilocular cystic lesion measuring 20.0 × 15.0 cm, located in the right lobe of his liver. It was associated with compression over the porta hepatis with mildly prominent intrahepatic ducts in both lobes of his liver (Fig. 4). A radiological diagnosis of giant hydatid cyst resulting in obstructive jaundice secondary to compression effect was made. Surgical excision was performed in December 2015. The specimen comprised a single, tan brown, irregular, flattened tissue piece measuring 7.5 × 6.5 cm. The wall thickness was 0.4 cm. The outer surface was smooth while the inner surface was irregular and hemorrhagic. Solid and nodular areas were not seen. Multiple cystic spaces were seen. Histopathologic examination showed a cyst wall lined by a single layer of tall columnar epithelium with basally placed nuclei and apical mucin (highlighted on PAS+/−AB). Dysplasia was not seen. Underlying stroma was fibroconnective and did not look like ovarian-type stroma. Fibrinopurulent exudate was also seen (Fig. 5). A diagnosis of intrahepatic biliary cystadenoma was made. Surgical excision was incomplete as the cyst was 7.5 × 6.0 cm while radiologically the complete cyst measured 20.0 × 15.0 cm. The patient has so far not undergone a repeat resection for removal of residual tumor.

## Discussion

There is currently no specific diagnostic method for the preoperative diagnosis of these tumors. Enhanced CT is the main preoperative diagnostic method used and shows internal septations, as well as papillary projections and/or mural nodules in some cases. However, the preoperative diagnostic rate is still quite low [17]. These tumors are often misdiagnosed as hydatid cysts or simple cysts of the liver. This often leads to incomplete excision. It is important to emphasize that in those cases where these tumors have been clinically and/or radiologically misdiagnosed as hydatid cysts, careful and complete removal of the cyst with cystic fluid is essential as rupture of the cyst wall during surgery may lead to leakage of cyst contents into the body and may result in anaphylactic shock. However, the most common treatment for hydatid cysts of the liver is a conservative surgery which involves the suction of the cyst contents with sterilization of the cavity, together with partial cyst resection [18, 19]. At other times, they are treated by laparoscopic fenestration. In contrast to hydatid cysts, incomplete or subtotal cyst excision or treatment of these cystic tumors by laparoscopic fenestration results in early recurrence [5–7, 17, 20]. It must be remembered that the presence of internal septations on a preoperative CT scan is highly suggestive of intrahepatic biliary cystadenoma [5]. As these tumors are rare and give rise only to nonspecific symptoms such as abdominal pain and fullness, clinicians often do not think about these tumors and are sometimes not even aware of them, which leads to incorrect clinical diagnosis as simple or hydatid cysts [17]. This is often disastrous for the patient because misdiagnosis results in inadequate treatment. Unfortunately, these tumors have a high recurrence rate and an estimated 20% undergo malignant transformation if incompletely excised [20]. According to many authors, an aggressive surgical approach in the form of complete radical excision with free margins is essential to prevent early recurrences [5, 6, 19, 21]. However, other authors recommend a less aggressive approach such as enucleation with free margins or segmental liver resection [5, 7]. According to one study, enucleation with free margins is an option in cases where resection is not possible [21]. Radical resections of the liver are complicated surgical procedures which are technically demanding and need to be performed at specialist centers in order to minimize complications [9]. In our countries, surgical expertise regarding liver resections is very limited. The high recurrence rate of biliary cystadenomas in our series is reflective of incomplete excision both because of incorrect preoperative diagnosis and lack of expertise in liver surgery. It is essential to think of these tumors in the differential diagnosis of cystic lesions in the liver especially if a CT scan reveals internal septations and even more so if the patient is a woman. In our series, five out of six patients were women and many newer studies have confirmed the female preponderance of these tumors [7, 17, 21]. It must be also remembered that sometimes these tumors can assume giant proportions [14, 20]. Occasionally, these rare tumors can give rise to even more unexpected clinical presentations such as acute pancreatitis due to compression of the pancreas [22], ascites with or without peritonitis due to spontaneous intraperitoneal rupture [23], varicocele and so on [24]. Pathologists can misdiagnose these cysts as simple cysts or hydatid cysts if they fail to concentrate on the cyst lining and ovarian-type stroma. Another differential diagnosis, which needs to be considered, is ciliated foregut cyst that is lined by ciliated epithelium but can also show a pericystic smooth muscle layer. The pathologist should also rule out this entity. In addition, the pathologist needs to be alert to the presence of moderate to high grade dysplasia in these cases and report when present. Recurrence occurred in three out of the five cases in our series in which follow up was available.

The World Health Organization (WHO) has recently recommended the term “mucinous cystic tumors” for intrahepatic biliary cystadenomas. However, a recent study argued that since the large majority of these tumors have a clear rather than mucinous fluid in their lumina and since the cysts are lined by a single layer of cuboidal to columnar epithelium, which more closely resembles the lining of the gallbladder and bile ducts rather than the lining of the intestine, the term “mucinous cystic tumor” is a misnomer. Thus, the terminology and epithelial phenotype of these rare tumors may remain controversial even in the near future [25]. As ovarian stroma is not seen in some of these tumors in males, a recent paper has suggested that such tumors be classified as “Intraductal papillary mucinous neoplasms, biliary type with marked cystic elements” [21].While it is important that every effort be made for correct preoperative diagnosis of these rare tumors, surgery followed by histological examination still remains the only definitive means of an accurate diagnosis [26].

## Conclusions

Pathologists need to: ensure adequate sampling of the specimens, especially from any solid or nodular areas; be careful to document the presence of moderate to high grade dysplasia in the histologic sections; and to keep the main differential diagnoses including hydatid cyst of the liver, simple cyst, and ciliated foregut cyst in mind.

## Abbreviations

AKUH: Aga Khan University Hospital
ASMA: Anti-smooth muscle actin
CBD: Common bile duct
CK: Cytokeratin
CT: Computed tomography
ERCP: Endoscopic retrograde cholangiopancreatography
H&E: Hematoxylin and eosin
IHC: Immunohistochemical
IVC: Inferior vena cava
PAS+/−AB: Periodic acid–Schiff +/− Alcian blue
WHO: World Health Organization
